# Food Banks against Climate Change, a Solution That Works: A Case Study in Navarra, Spain

**DOI:** 10.3390/foods11223645

**Published:** 2022-11-15

**Authors:** Josemi G. Penalver, Alejandra Armijos, Beatriz Soret, Maite M. Aldaya

**Affiliations:** 1 School of Agricultural Engineering and Biosciences, Public University of Navarra (UPNA), Arrosadia Campus, 31006 Pamplona, Spain; 2 Institute for Sustainability & Food Chain Innovation (IS-FOOD), Public University of Navarra (UPNA), Arrosadia Campus, 31006 Pamplona, Spain

**Keywords:** sustainability, food waste, carbon footprint, greenhouse gases, climate change

## Abstract

Worldwide, more than 1.3 billion tonnes of food are wasted each year, which is equivalent to releasing 4.4 Gt of CO_2_ equivalents (CO_2_e). In this context, the Food Bank of Navarra (FBN) annually avoids the waste of approximately 3000 tons of perfectly consumable food. The aim of this study was twofold: on the one hand, to analyse the carbon footprint of the FBN and, on the other hand, to perform a comparative analysis of the greenhouse gas (GHG) emissions in two scenarios, “with” and “without” the actions of the FBN, in order to identify and quantify the environmental benefits, in terms of GHG emissions reduction, associated with the reduction in food waste. The analyses were conducted in two different years. The carbon footprint associated with the FBN’s activities was 147 t of CO_2_e in the year 2018. The quantification of GHGs in the scenario “without the FBN” showed that if the FBN did not exist 4715 t of CO_2_e would have been emitted. The results obtained in consecutive years were similar, highlighting the importance of the FBN—not only in social terms but also environmental terms—as it prevented a large amount of GHGs from being emitted into the atmosphere. A detailed account of the carbon emission reduction associated with the food bank’s operations and the knowledge of the benefits involved could boost their positive effects in facilitating the integration of their activities into policies aimed at climate neutrality.

## 1. Introduction

The amount of food that is produced but not used by consumers is extremely high, reaching more than 1.3 billion tonnes per year worldwide [1]. This is a significant issue, as this type of food waste represents one of the largest inefficiencies in the global food system. There are, nevertheless, different definitions of food loss and waste related to the complexities of the various food supply chains [2]. The most commonly accepted definition is the one provided by the Food and Agriculture Organization of the United Nations, which defines food loss and waste as a decrease in the quantity or quality of edible food along the food supply chain. Food losses often occur in the first stages of the supply chain, including the production, postharvest, and food processing stages; however, this definition does not include the inefficiencies or losses at the retail level. The definition of food waste, on the other hand, includes losses at the retail and consumption levels [3]. Between the different strategies to reduce food waste are actions that pursue consumer awareness of the problem, behavioural change, and actions related to food donation [4].

According to the Food Waste Index report 2021 [5], around 931 million tonnes of food waste were generated in 2019, 61% of which came from households, 26% of which came from food service, and 13% of which came from retail. This suggests that 17% of total global food production may be wasted (11% in households, 5% in food service, and 2% in retail). 

Beyond the socioeconomic issues that food waste poses, there is an increasing amount of literature highlighting how food waste leads to the waste of fuel, energy, agrochemicals, water, land, and the labour that are all invested in its production [6,7,8]. A relation between food waste and negative environmental impacts has been demonstrated, such as water resource depletion, photochemical ozone formation, water eutrophication, human toxicity, fossil resource depletion, land acidification, particulate matter, ecotoxicity, and global warming [9,10,11,12,13,14]. Food production is a resource-intensive activity and a significant contributor to GHGs. The food system is connected to around 35% of the total global anthropogenic GHG emissions [15]; further, global food waste represents about 8% of the total anthropogenic GHG emissions, i.e., 4.4 Gt CO_2_e [1]. As such, if food is wasted, it entails negative impacts on global warming [16]. At a regional level, according to the Spanish Ministry of Agriculture, Fisheries, and Food, Spain is the country in the European Union with the seventh highest quantity of food waste (7.7 million tonnes) [16].

The concern regarding the stabilisation of greenhouse gas (GHG) concentrations in the atmosphere has been growing in recent years. Achieving lower levels of anthropogenic interference would prevent further temperature increases and, therefore, unwanted changes in the global climate system. Unfortunately, this goal has not been achieved, and the loss of this atmospheric equilibrium has been causing a slow but steady rise in global average temperatures [17].

In this context, food banks can contribute to mitigating these problems, as their activities prevent the waste of perfectly consumable food that would otherwise be wasted. In particular, the Food Bank of Navarra (FBN), in Spain, avoids wasting an average of 3000 tonnes of food every year. It is important to remark, however, that its mission, as a basis for its integration, is not only to improve the food security of the groups of people who are at risk (or in social exclusion and/or in a poverty situation) but also to involve society and the public authorities in this process [18]. 

Different methodologies have been developed in order to evaluate the impact of GHG emissions derived from anthropogenic activities with regard to climate change. The most standardised and widely used method is the carbon footprint, defined as the total amount of GHG emissions that are produced and released to the environment as a consequence of performing an activity [19]. 

There are a few studies that have quantified the GHG emission savings—among other positive environmental impacts—of surplus food redistribution (which are related to the retail sectors, supermarkets, and food service sectors in different countries [20,21,22,23,24,25]). These studies accounted for the GHG emissions that are related to different hypothetical scenarios, including prevention, redistribution, animal feed, and different food waste management options. Among those studies, the ones including donations of food waste to charity organisations simply included the environmental impact of transport without considering the impact of the charitable organisation itself [20,23,24]. There are differences in the specific results obtained by each study that can be explained by the differences related to the system boundary settings, the assumptions made, and the variations in input data contained in the respective studies [26]. Overall, these studies showed a waste hierarchy, ranking food prevention and donation as the best options, while landfilling was one of the least beneficial alternatives. 

Finally, just two studies have specifically focused on quantifying the positive impacts on climate change, among other environmental impacts, of food rescue by charitable organisations [27,28]. The study published by Reynolds et al. [27] quantified the positive environmental and economic impacts of food rescue at the national level in Australia using an extension of the waste input–output framework. According to their study, every US dollar spent on food rescue represented 7.5 kg of embodied greenhouse gases (CO_2_ equivalents) related to food that was redirected from being sent to landfill or composting and was sent to food-insecure individuals. However, the environmental impact values of the study by Reynolds et al. did not include the resources used or the GHG emissions generated as part of the food rescue operation. Damiani et al. [28] analysed the carbon footprint of 1 kg of redistributed food for Italian solidarity emporiums using a life cycle analysis (LCA) approach. Food donation reduced the average impact of the studied systems with a 1.9 kg CO_2_ eq/kg net environmental benefit. Nevertheless, they did not assess the carbon footprint at the organisational level. To our knowledge, there are only two nonpublished technical reports about this topic, which were applied to the food banks of Bordeaux, France [29] and Córdoba, Spain [30]. However, there are no published scientific papers that account for the positive influence of food banks at the organisational level in reducing GHG emissions while also considering, in a comprehensive fashion, the environmental impacts of the charity organisations themselves. This approach, therefore, poses a challenge in the conceptualisation of the scenario model to be applied as well as the challenge of overcoming the constraints related to the application of a life-cycle approach, such as the source of emission factors and data collection.

The present publication quantifies, for the first time, the impact of the activities of a food bank related to GHG emissions in two different years using a life-cycle approach. First, this work assessed the carbon footprint generated by the activities of the Food Bank of Navarra (i.e., a scenario with the existence of the FBN). Second, it analysed the GHG emissions in a theoretical scenario without the FBN, considering the emissions released due to waste management and additional food production (i.e., a scenario without the existence of the FBN). Subsequently, this work focused on a comparative analysis of the two scenarios—with and without the FBN’s actions—thereby identifying and quantifying the environmental benefits in terms of GHG emissions reduction as well as those benefits that were associated with the reduction in food waste. The results for the year 2018 were then compared with those of the year 2019 to ensure the reliability of the results. As a conclusion, the study confirmed the climate change mitigation effects of this type of organisation.

## 2. Materials and Methods

### 2.1. Greenhouse Gas Emission Assessment and Methodology 

The assessment of GHG emissions for the years 2018 and 2019 were performed according to international standards [19,31,32,33,34]. A life-cycle assessment approach was used for the purposes of assessing the GHG emissions that were associated with the activities of the FBN, i.e., covering all the stages of the development of the activities, from the extraction of raw materials, processing, manufacturing, and distribution to the use and the end-of-life stages (deposit, reuse, or recycling). In this manner, it was possible to allocate the amounts of GHG emissions in the stages and to, therefore, identify those stages where the largest amounts of emissions were generated [19].

For the determination of GHG emissions, the following steps were applied: (A) the definition of boundaries; (B) the identification of emissions; and (C) the calculation of emissions [19].

#### 2.1.1. Boundary Definition

In this first phase, the system’s organisational and operational boundaries were established; in addition, the types of emissions to be included in the inventory were determined. 

The emissions were classified in the following categories: (1) direct or “scope 1” emissions, which consider all GHG emissions that come from sources that are owned and controlled by the organisation; (2) indirect or “Scope 2” emissions, which are emissions that are specifically associated with the consumption of electricity and energy purchased by the organisation; and (3) “other indirect” or “Scope 3” emissions involve the indirect emissions related to subcontracted services, purchased products, and transport as well as others that are not included in scopes 1 and 2. 

#### 2.1.2. Identification of Emissions

In a second step, an inventory of all emissions generated at each working facility was identified and drawn up, differentiating between scopes 1, 2, and 3.

#### 2.1.3. Calculation of Emissions

The GHG emissions (E, quantity of CO_2_e/time) were calculated by multiplying the activity data (AD, quantity of units/time) by the emission factors of the activities (EF, mass of CO_2_e/unit); the GHG emissions are generally expressed in tonnes of CO_2_ equivalent (tCO_2_e) [35].

(1) $$E=\mathrm{AD}\times \mathrm{EF}\;[\mathrm{Quantity}\;\mathrm{of}\;{\mathrm{CO}}_{2}e/\mathrm{time}]\;$$

Total GHG emissions were calculated by applying this equation to the different activities (subtotals) for scopes 1, 2, and 3 and then adding all the subtotals.

### 2.2. Scenario with the Food Bank of Navarra 

The Food Bank of Navarra is an independent nonprofit organisation that was created in 1996. Located in the Spanish autonomous community of Navarre, it acts as an intermediary between industries and organisations that have surplus food and the social entities in charge of distributing supplies to groups who are at risk of poverty.

At the time of the study, the FBN had two offices: one in Berrioplano, which attended to the needs of the Pamplona region, and another in Tudela. It was managed by 176 permanent volunteers, of whom 160 worked in Berrioplano and 16 worked in Tudela. Briefly, the FBN’s operations consisted of receiving, classifying, and storing food for its subsequent distribution.

The food came from multiple sources. The most important of these are listed below:

- Agrifood industries: approximately 104 donor companies were responsible for supplying food to the FBN.
- Fruit and vegetable producer organisations (FVPOs): within the framework of the regulation of Spanish markets, the FVPOs delivered fresh fruit and vegetables to all food banks.
- The Pamplona Region Commonwealth (PRC): This organisation collected foods from 67 shops as well as from 15 distribution companies on a daily basis, which was all perfectly consumable. These foods were donated because they had an early expiry date or the packaging containing them was defective, which classified them as unmarketable foodstuffs.
- Twice a year, solidarity campaigns called Grandes Recogidas (large food collections) were organised, which consisted of collecting food that was requested from the general public and from distribution companies.
- European Aid Fund for the Most Deprived: this programme consisted of the purchase of food on the market through a public tendering procedure for its subsequent supply to partner distribution organisations, in this case the FBN [36].
- Other food banks: the FBN helped, in 2018 alone, with the redistribution of 93 tonnes of food received from 19 food banks that were located throughout Spain.

As mentioned above, the food collected by the FBN was distributed to accredited social entities in the autonomous community of Navarre. In 2018, a total of 3748 tonnes of food was distributed through 193 entities in Navarre, benefiting 24,652 users.

**Figure 1:**
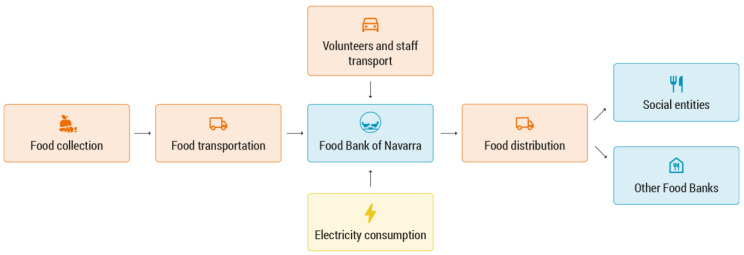
Activities considered in the scenario with the Food Bank of Navarra (FBN). In this scenario, the food collection points included: manufacturers and distributors, The Fund for European Aid to the Most Deprived, food collections, the Pamplona Region Commonwealth, fruit and vegetable producer organisations, and other food banks and food donations [12].

This scenario accounted for the GHG emissions directly caused during the FBN’s daily activities, which included the collection of food; the transport of incoming food; the transport of volunteers and hired staff; reception; the classification and storage of food in the FBN headquarters; and its distribution to social entities. The FBN had two headquarters: the main one located in Berrioplano and a secondary one in Tudela. An overview of the FBN’s activities with the associated emission sources can be found in Figure 1.

The quantified emissions were collected as follows:

“Scope 1” emissions were the direct GHG emissions that came from the combustion of natural gas for the purposes of heating at the main FBN headquarters as well as the emissions from powering the vans owned by the FBN.

“Scope 2” emissions were indirect emissions associated with the generation of electricity purchased and consumed at the facilities of the FBN.

“Scope 3” emissions were other indirect emissions caused by the transport of the rescued food from donors to the FBN, the transport of food from the FBN to social entities, the travel of volunteers and hired staff, and the purchase of products and services by the FBN (cardboard, wooden pallets, and the drinking water supply).

The activity data were provided directly by the FBN. These data came from consumption invoices, vehicle mileage, interviews of volunteers and hired staff, and food records coming into the FBN. Emission factors were obtained from internationally recognised sources of information (see Supplementary Material, Tables S1–S10). 

### 2.3. Scenario without the Food Bank of Navarra 

**Figure 2:**
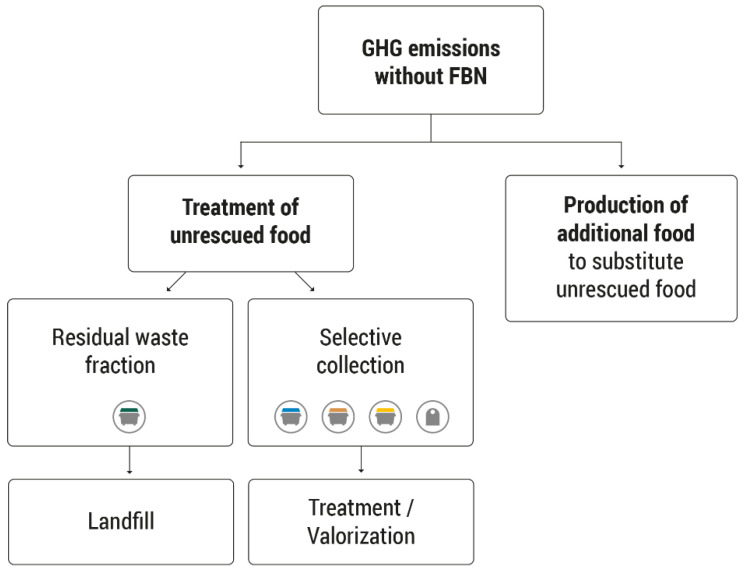
Activities considered in the scenario without the Food Bank of Navarra (FBN) [12].

The hypothetical scenario without the FBN followed the system boundaries used in similar studies [29,30]. In this theoretical scenario, most of the food that was recovered by this institution would be wasted. Therefore, the absence of the FBN would entail the emission of GHGs as a result of two elements (Figure 2):

(a) The fate of food not distributed by supermarkets or other producers and suppliers. Food waste, mostly organic, would be generated, and the management of this waste would be necessary. This waste, depending on its nature and local management system, would be deposited in controlled landfills or undergo treatment or recovery, which would entail more GHG emissions to the atmosphere.

(b) The need for the FBN beneficiaries to be fed in the absence of the FBN. It would be necessary to produce additional food as a replacement for the food that was not rescued. In this case, it was assumed that the same food products donated to the food bank would be reproduced with the related GHG emission release. 

In this same scenario, i.e., “without the FBN action”, only donor entities that avoid food waste were considered, such as surplus donated by agrifood companies (manufacturers and distributors); fruit and vegetables from the withdrawal of surplus by the fruit and vegetable producer organisations; nonmarketable consumable food withdrawn by the Pamplona Region Commonwealth on a daily basis; and products from other food banks.

#### 2.3.1. Additional Food Production Data Collection

The FBN distributed, in 2018 alone, around 2.7 thousand tonnes of consumable food. In a scenario without the FBN, this food would be wasted, and new food would be necessary to feed the FBN beneficiaries. In order to understand the impact that this additional food production would entail to the environment (in terms of GHG emissions), it was assumed that the same food would be reproduced. 

For this purpose, the products entering the food bank were classified and quantified via being assigned a category, which, according to the database provided by the FBN, included baby food, unclassified food, beverages, pastries, biscuits and sweets, cocoa and chocolate, coffee and teas, meats, cereals, flour, bread and pasta, condiments and sauces, canned vegetables, fruit, canned fruit, dried fruit, vegetables and pulses, eggs, fish, ready meals, dairy products, and assorted snacks.

Subsequently, the GHG emission factors associated with each type of product were taken from the ADEME database [37] as well as, complementarily, from ECODES [38], the report of Guilhem [29], and the Cátedra de Ética Ambiental [39]. The emission factors from the ADEME database possessed an uncertainty of 30%. In this database, the emission factors for each agricultural product covered the range from the cradle to the gate of the field or facility. For plant products, this included all input manufacturing processes and field operations but excluded postharvest processes (e.g., storage, drying, etc.). For animal products, this database included all on-farm processes (livestock, storage, the manufacture of livestock feed on the farm, milking parlour and milk tank operations, etc.). In-store agrifood products included emissions associated with plant and animal products; processing stages; packaging production; and transport and storage. Further, this database did not include GHG emissions related to the transport of consumers between their homes and the shops, secondary packaging production, waste management, and end-of-life packaging [37]. Therefore, the GHG emissions results for each food product were slightly underestimated.

The details of the food categories and the quantities that were managed by the FBN, together with their activity data and the emission factors associated with the additional food production, are summarised in the Supplementary Material, i.e., Table S11.

#### 2.3.2. Waste Treatment Data Collection

In order to identify the sources of GHG emissions associated with the waste management of food that was not consumed and was not rescued by the FBN, the locations of the organisations from which the products originated were first identified; in addition, these locations were then further divided into three different geographic zones. Then, the amount of food that would be wasted per zone was quantified. Thereafter, the waste management systems most used in each geographic territory were determined. Finally, the GHG emission factors for each management system were collected.

The different geographic areas from which the donated food came included:

- The Pamplona Region Commonwealth: 778 tonnes;
- The sources of food supply to the FBN that were located in the PRC’s area included: the surplus food donated by agrifood companies (i.e., manufacturers and distributors) (54%); the daily PRC collection for the FBN (41%); and the surplus food from the fruit and vegetable producer organisations (5%).
- The percentages of the different waste fractions were based on the specific data from the PRC, published in the 2018 “Inventory of household and commercial waste” of the Government of Navarra [40]; this was in addition to the study regarding the characterisation of household waste in 2018, which was carried out by the Office for Waste Prevention and Promotion of the Circular Economy of the PRC [41].
- The rest of Navarra: 853 tonnes;
- In the rest of Navarra, excluding the PRC, the source of donated food was the surplus food donated by agrifood companies (manufacturers and distributors). The percentages of the different waste fractions were based on the data from the Government of Navarra [40].
- The rest of Spain: 1136 tonnes. Finally, the sources supplying food to the FBN from the rest of Spain included the surpluses donated by FVPOs (71%), the agrifood companies (manufacturers and distributors) (21%), and other food banks (8%).
- Of all the autonomous communities that were consulted, only the Generalitat de Cataluña provided the details of the final waste treatment. Due to this lack of publicly available information in most of the Spanish Autonomous Communities, it was assumed that these companies followed a similar waste management pattern to that of the Navarran (excluding PRC) and Catalan donor companies. In other words, for the “rest of Spain”—with the exception of Catalonia—the averages of the final waste treatments of the Comunidad Foral de Navarra and the Generalitat de Cataluña were assumed.

The emission factors associated with the valorisation or disposal processes were selected according to the most recent literature. The factors for biomethanisation and the composting of organic matter were taken from the National Greenhouse Gas Inventory of the Spanish Ministry for the Ecological Transition and the Demographic Challenge [42]. On the other hand, the emission factors for the final treatment of paper, cardboard, light packaging, and glass were taken from the report on the “Calculation of GHG emissions from municipal waste management” from the Catalan Office for Climate Change [43]. In the case of emissions from the Góngora Waste Treatment Centre, which managed the PRC waste, the factors from the PRC annual carbon footprint report were used (personal communication from Álvaro Miranda, from the PRC, 16 April 2020).

The details of the food waste systems and the quantities of food waste managed by the FBN, together with their activity data and the emission factors associated with the waste of food, are summarised in the Supplementary Material, i.e., Tables S12–S17.

### 2.4. Description of the Carbon Balance: A Comparison of the Emissions with and without the Actions of the Food Bank of Navarra

A comparative analysis between the scenario with and without the actions of the FBN was carried out in order to determine the positive or negative effects of the actions of the food bank on GHG emissions as well as the effects related to global warming. This analysis contrasts the situation that occurred in 2018, i.e., with the FBN operating normally, with the hypothetical scenario that detailed a situation without the existence of the FBN.

It should be noted that some activities that were associated with GHG releases may have occurred in both scenarios, for example, the emissions caused during the waste management of the packaging and the partially consumed food, the emissions generated during the cooking and processing in the consumer phase [44], and the emissions generated in the transport of food products from the food distribution organisations to the consumers’ homes. Although these types of activities would indeed cause the emission of GHGs, the balance would not be affected, as the same emissions would take place in both scenarios (with and without the FBN). In other words, they were not variables; but features that were the same in both situations. Therefore, on this basis, these emissions were not taken into account.

## 3. Results 

### 3.1. Greenhouse Gas Emissions in the Scenario with the Food Bank of Navarra

#### 3.1.1. Carbon Footprint of the Food Bank of Navarra

**Figure 3:**
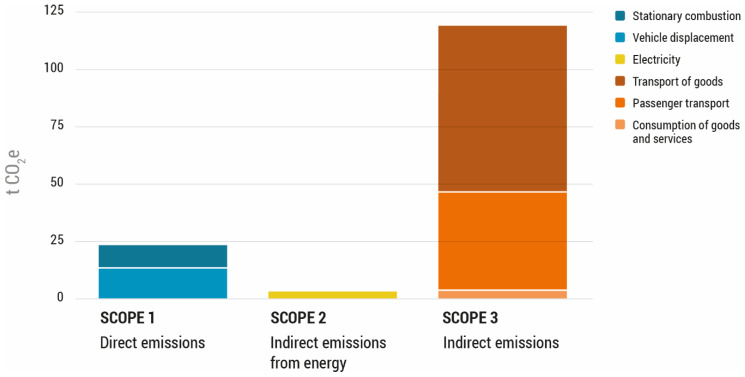
Greenhouse gas emissions in 2018 from the activities of the Food Bank of Navarra, detailed according to certain scopes (in tonnes of CO_2_e).

The carbon footprint of the FBN’s activities was 147 tonnes of CO_2_e in the year 2018. The major emission sources corresponded to scope 3 (indirect emissions), which were 81% of the total emissions of the FBN. The main contributor to scope 3 was the fuel consumption that was required for the transport of the donated food, releasing a total of 72,744 kg of CO_2_ eq. Secondly, GHG emissions generated by scope 1 (direct emissions) accounted for 17% of the total emissions, whose main source of emissions was the transport of staff. Finally, scope 2 (indirect emissions specifically associated with electricity and energy consumption) accounted for 2% of the total FBN emissions and were entirely caused by electricity consumption. The emissions of each scope are illustrated in Figure 3.

It should be noted that there were no emissions due to refrigerant gas recharge (scope 1). For the energy provided by other energy suppliers in the other facilities of the FBN, the corresponding mix of energy sources was used.

Detailed results regarding energy sources, conversion factors, and sources of information can be found in the Supplementary Material, i.e., Tables S1–S10.

#### 3.1.2. Carbon Reduction Plan

As indicated, the transport of goods as well as volunteers and staff were the main source of GHG emissions, as they represent around 79% of the total emissions of the FBN. In order to reduce the emissions from this source, different options were analysed. Promoting the use of public transport, carpooling, or walking among the volunteers and staff hired by the FBN would all be effective ways to reduce the organisation’s carbon footprint. The use of the city bus as a means of transport for 25% of the staff who drive to the FBN was also proposed. This would help to reduce the GHG emissions associated with staff travel. However, for this option to be feasible, there would need to be an improvement in the public transport links to the FBN, as the total commuting time required from different points in Pamplona is currently very high, especially when taking into consideration a last stretch that is required on foot. Cycling or using electrical scooters were not considered as options, as most of the FBN volunteers were retired people.

It was also advised to make better use of renewable electricity in all the facilities of the FBN. The Tudela headquarters could contract a renewable electricity service, such as in the Berrioplano headquarters, as this type of energy does not emit meaningful amounts of GHGs, according to [45].

The reductions that could be reached once the improvement plan was implemented, assuming that the level of activity remained at the same level as in the base year of 2018, was estimated. Therefore, the reductions would be a consequence of improved efficiency. The results, translated into terms of carbon reduction, are shown in Table 1. As can be observed, these changes, when including a change to renewable energy use and a 25% increase in public transport, would reduce carbon emissions by 9%.

### 3.2. Greenhouse Gas Emissions in a Scenario without the Food Bank of Navarra

**Figure 4:**
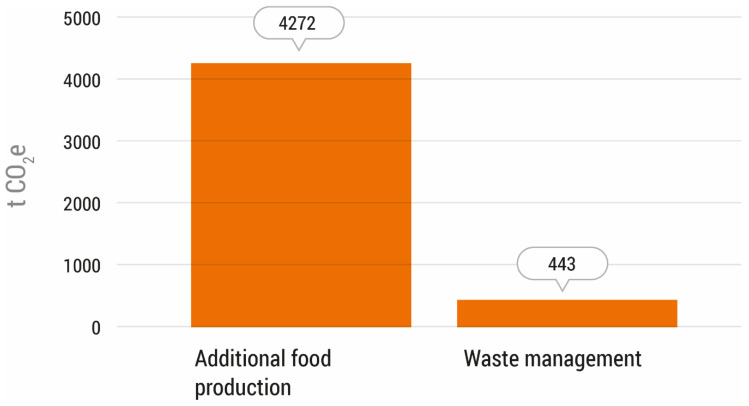
Greenhouse gas emissions by category in the scenario “without the action of the Food Bank of Navarra” in the year 2018 (tonnes of CO_2_e).

In the theoretical case where the FBN did not exist, the GHG emissions would have reached a total of 4715 tonnes of CO_2_e in 2018, of which 91% would have been emissions associated with additional food production, while 9% would have been emissions associated with waste management (Figure 4).

#### 3.2.1. GHG Emissions from Additional Food Production 

In a scenario without the actions of the FBN, the GHG emissions related to the additional food production of 2767 tonnes of food would have reached 4272 tonnes of CO_2_e.

From the 20 food categories that were considered, 11 types of food would have generated the highest GHG emissions, accounting for 98% of the total emissions. The foodstuffs that would generate the most GHG emissions would be dairy products, amounting to 896 tonnes of CO_2_e, which would represent 21% of the total emissions in this scenario, followed by vegetables and legumes (12.1%), ready meals (11.6%), fruits (10.9%), and canned vegetables (7.4%).

The details of the food categories and quantities managed by the FBN, together with the associated emissions related to the additional food production, are summarised in the Supplementary Material, i.e., Table S18.

These results have an uncertainty of about 30%, as the data available for the calculation of emissions from food production were scarce and most emission factors were taken from the ADEME database [37]. The obtained results cover the scope from the stages of input manufacturing, field operations, emissions from livestock facilities, processing, packaging production, transport, and storage, but do not include GHG emissions related to the transport of consumers between their homes and the places of purchase or the production of secondary packaging. Therefore, the GHG emissions results that are presented for each food are approximate and possibly underestimated.

#### 3.2.2. GHG Emissions from Waste Management 

The GHG emissions associated with the management of the 2767 t of food that would be wasted in this scenario were estimated to be 443 tonnes of CO_2_e. This type of waste is sent to controlled landfills or to valorisation treatments, such as biomethanisation or composting in the case of organic matter, recycling treatments, or reuse (in the case of paper, cardboard, light packaging, and glass). 

**Figure 5:**
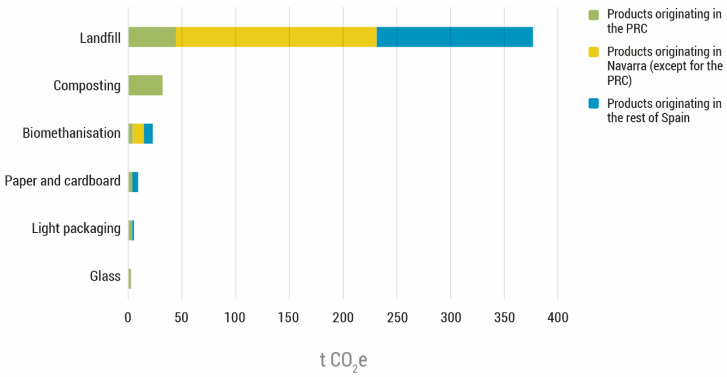
Greenhouse gas emissions in 2018 associated with waste management, organised by the type of treatment and location in the scenario “without the action of the Food Bank of Navarra” (in tonnes of CO_2_e).

Of the management options that were analysed, landfilling would account for 85% of the total emissions generated by waste management (Figure 5). These emissions correspond specifically to the final treatment of waste but do not include the emissions generated by the transport of waste. This likely led to a slight underestimation of the emissions.

### 3.3. Comparative Analysis of GHG Emissions “with” and “without” the Activities of the Food Bank of Navarra

The total GHG emissions for the year 2018 “with the actions of the FBN” amounted to 147 tonnes of CO_2_e. In a scenario “without the actions of the FBN”, the emissions that would have been generated by waste management and additional food production would have been much higher, reaching 4715 tonnes of CO_2_e.

**Figure 6:**
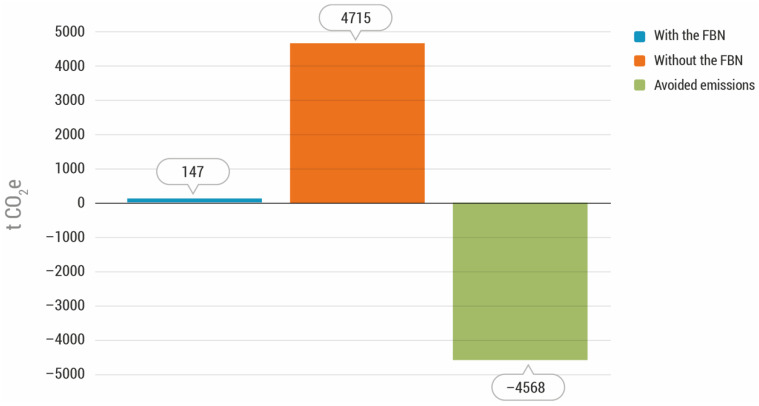
Total greenhouse gas emissions in the scenarios “with” and “without” the actions of the Food Bank of Navarra in 2018 as well as the emissions avoided by the Food Bank of Navarra (in tonnes of CO_2_e).

Therefore, the carbon footprint of the FBN in 2018 could be qualified as “negative”, as its activity prevented the emission of 4568 tonnes of CO_2_e (Figure 6). In other words, the actions of the FBN reduced GHG emissions by 97% compared to a theoretical scenario without the actions of the FBN. The balance of the GHG emissions associated with the activity of the FBN was, environmentally, very positive when understood in terms of its contribution to the reduction in global warming.

### 3.4. Verification of the Results in Two Different Years

#### 3.4.1. Scenario with the Food Bank of Navarra 

The carbon footprint of the FBN’s activities increased by 0.8 tonnes of CO_2_e between 2018 (146.9 t CO_2_e) and 2019 (147.7 t CO_2_e), representing an increase of 0.6%. This increase in GHG emissions was reflected in scope 1 due to an increase in the consumption of natural gas for heating at the FBN facilities and in scope 3, mainly due to an increase in the fuel consumed for the transport of people, i.e., 0.6 and 0.5 t of CO_2_e, respectively. However, there was a decrease in scope 2 emissions, i.e., 0.5 fewer tonnes of CO_2_e in 2019 compared to 2018. This was due to a decrease in the emission factor of the energy mix used by the energy supplier (see Supplementary Material, Figure S1).

#### 3.4.2. Scenario without the Food Bank of Navarra

The GHG emissions in a hypothetical scenario “without the actions of the FBN” would have been 9% lower in 2019 (4304 t CO_2_e) compared to 2018 (4715 t CO_2_e). This is because in 2019 the FBN collected and distributed 333 fewer tonnes of food than in 2018, 2434 tonnes vs. 2768 tonnes, respectively. Thus, the emissions associated with the additional food production and waste management would also be lower.

In both 2018 and 2019, the additional food production would have accounted for approximately 91% of the total emissions in this scenario, while emissions associated with waste management would have been close to 9% (see Supplementary Material, Figure S2).

#### 3.4.3. Comparative Analysis with and without the Actions of the Food Bank of Navarra

In both cases, 2018 and 2019, the emissions generated in a scenario “without the actions of the FBN” would have been significantly higher than those associated with the carbon footprint of the Food Bank of Navarra: 4715 vs. 147 t of CO_2_e in 2018 and 4304 vs. 148 t of CO_2_e in 2019. In other words, in both years the activity of the FBN prevented the emission of huge amounts of GHGs that were associated with food waste. Thanks to the FBN’s activity, around 4568 tonnes of CO_2_e were not released to the atmosphere in 2018 and around 4157 tonnes of CO_2_e were not released to the atmosphere in 2019. In both cases, the actions of the FBN reduced GHG emissions by 97% when compared to the scenario “without the actions of the FBN” (see Supplementary Material, Figure S3).

## 4. Discussion 

### 4.1. Research Results Comparison with Previous Studies

The present study shows the positive actions of the Food Bank of Navarra in terms of reducing food waste and the associated greenhouse gas emissions, which is in line with the results of similarly structured previous studies (Table 2). However, slightly different outcomes were obtained by the two available previous studies, which were most likely due to the differences in the applied methodologies and the quantities of food distributed annually.

As shown in Table 2, the study on the Food Bank of Cordoba [30] obtained lower GHG emission savings in the final comparative balance. This was due to the fact that only around 9% of the food that the food bank managed annually was donated by food companies. The remaining food came from consumer donations, which did not necessarily mean that food waste was avoided. Furthermore, the study on the Food Bank of Cordoba did not take into consideration the emissions generated by the valorisation and disposal treatments. 

Conversely, the study on the Food Bank of Bordeaux [29] showed by far the largest GHG emission savings in the comparative balance (Table 2). This makes sense, as the quantity of food distributed by this bank was much larger than the quantity distributed by the FBN. The study focused on the Food Bank of Bordeaux also included the impact generated by the waste management of the food consumed by the food bank users in both scenarios. These emissions were not included in our study; this was due to the fact that the result would be the same for both scenarios (with and without the FBN), and they would, therefore, offset each other in the comparative analysis. Furthermore, for the final carbon balance, this report took those GHG emissions that were avoided due to the use of appropriate recycling and valorisation treatments and compared them to the emissions that would be released without appropriate waste management. In our opinion, this has significant value in a conceptual sense but is rather inaccurate when considering the actual carbon footprint generated by such an organisation.

Another study, conducted at the product level by Damiani et al. [28], obtained similar results: food donations reduce the GHGs of the studied systems (1.9 kg CO_2_e/kg net environmental benefit). Their results revealed the importance of taking care of the size of emporiums, energy consumption, and logistics to optimise the redistribution process. They also particularly highlighted the relevance of the impact of logistics for surplus food collection. Most of the previously published studies simply took the environmental impact of the transport related to food redistribution into account [20,23,24]. The present study demonstrates the relevance of making a complete accounting of the environmental impacts of charitable organisations. For instance, in the case of the Food Bank of Navarra, it is crucial to consider not only the GHG emissions related to food transport (50% of the carbon footprint of the bank) but also the transport of volunteers and staff (30% of the carbon footprint of the bank), which has not been considered in any of the studies published so far in scientific journals.

Other studies focused on GHG emission savings in different scenarios also found the greatest potential for reducing emissions in food donations. This was found to be the case, even over animal feed and different food waste management options (landfill, incineration, composting, anaerobic digestion, etc.) [22,24]. After the food donation option, the best options were found to be anaerobic digestion, conversion to animal feed, incineration with energy recovery, aerobic composting, and landfill [22,23,24]. Among the analysed waste management systems, the current study also demonstrated the landfill option to be the worst one. Investigating the GHG emissions of different waste management options is important for the purposes of establishing a waste hierarchy in a local context [22].

### 4.2. Implications of the Study

The current study proved that food rescue by food banks helps to prevent more GHG emissions from being emitted into the atmosphere by employing a detailed carbon accounting framework. This finding, therefore, strengthens the environmental need for food waste reduction and food bank actions conducted at both the international and national levels. Global initiatives, such as the United Nations’ Sustainable Development Goals (SDG), include a target (12.3) for halving per capita global food waste at the retail and consumption stage by 2030 compared to 2015 [46]. The European Commission, in its Farm to Fork Strategy, also committed to halving per capita food waste at the retail and consumer levels by 2030, following SDG target 12.3. The Commission will promote coordinated actions at the European level, which will reinforce actions at the national level [47]. In addition, at the national level, the Spanish government has recently approved a first draft law that is intended to combat food waste [48]. According to this new law, each organisation that is part of the food chain must have a strategy in place to prevent food waste. Additionally, it establishes a hierarchy of essential objectives, with the first being the use of food for human consumption through contributions to charitable organisations or food banks [48]. To donate surplus food, the food value chain agents must sign collaboration agreements with companies, social entities, and other nonprofit organisations or food banks. In these agreements, the conditions for the collection, transport, and storage of the products, among other issues, must be specifically stated. The social entities that are the recipients of donations must guarantee the traceability of the donated products through a system designed for the purpose of registering the entry and exit of the food that is received and delivered. One of the reasons to promote food waste reduction along the food supply chain is that it would contribute to improved global food security and reduce the negative environmental effects brought on by the agrifood industry [49]. 

The actions of the Food Bank of Navarra in particular and of food banks as a whole can be considered a climate change mitigation measure. The last reports of the Intergovernmental Panel on Climate Change confirmed that the proposed measures to mitigate climate change have not been efficiently implemented so far and that this compromises the attempts to meet the challenges of the Kyoto Protocol and the Paris Agreement, which aim to prevent the global average temperature from rising by a maximum of 2 degrees Celsius [32].

The scientific community has been searching and proposing possible solutions to help reduce the emissions of anthropogenic GHGs in order to address this problem. Some examples, which provide solutions for both companies and consumers, offer ideas as simple as reducing food waste [50]; optimising irrigation and fertiliser application in agriculture; improving grazing management in livestock production [51]; selecting the appropriate dietary style [52] or being conscious of the origins of food; and utilising adequate use and end-of-life treatments that are needed for purchased goods [53]. Even so, there are options for those who are not willing to change their daily routines or their production processes, such as investing in external carbon mitigation projects [54].

Furthermore, the Food Bank of Navarra’s activity can also be considered a climate-offsetting scheme. One year of normal activity of the Food Bank of Navarra (2018, for example) would be equivalent to the fixation capacity of a forest area of 36 hectares in the north of Spain [45]. This would be in line with the Spanish Circular Economy Strategy and the Spain Circular 2030 of the Spanish Ministry for the Ecological Transition and the Demographic Challenge. Further, by knowing that the activity of the FBN helps to prevent the emission of an important amount of CO_2_e into the atmosphere, it could be considered that the FBN be registered into the CO_2_ sequestration projects section of the “Registry of carbon footprint, offsetting and carbon dioxide absorption projects”. This CO_2_ sequestration section allows for the establishment of offset project agreements between organisations that have registered their carbon footprints in the registry through CO_2_ absorption projects or through GHG emission reductions by a third party. In this sense, the FBN, as an organisation that reduces GHG emissions, could enter into agreements with organisations that wish to offset their carbon footprints. This option could be seen as a source of economic revenue, where a private company funds the food bank in order to offset its own carbon footprint, and therefore the FBN minimises its dependence on public resources.

### 4.3. Limitations of this Study

During the development of this study, a lack of public information on the types of management systems used by the different organisations was found (in our case, the types of management systems utilised by the donors to the FBN), which complicated the calculation of GHG emissions in a scenario without the activities of the FBN. 

In addition, the emission factors used for waste management corresponded specifically to the final treatment of waste but did not include emissions generated by the transport of waste. This likely led to an underestimation of emissions. Furthermore, the same types and proportion of use of waste treatments was assumed for 2018 and 2019, as only the total amount of waste managed per year was considered variable.

Regarding the determination of the carbon footprint of additional food production, the degree of uncertainty in these estimations is high, as there are uncertainties of 30% in the emission factors associated with food production. The same problem occurs with the determination of the GHG emissions associated with cardboard and wood production, which have uncertainties of 20 and 50%, respectively [37].

As the obtained data reflect a difference in emissions between the two scenarios of more than 4000 tonnes of CO_2_e and the range of error is most likely smaller, it is very likely that these uncertainties do not affect the conclusions obtained in this study about the positive impact on the environment by activity of the FBN.

## 5. Conclusions

The present article demonstrates how the activity of food banks is essential in terms of reducing food waste. Through its actions in 2018, the FBN helped 24,649 people to benefit from food that was going to be wasted. In particular, this contributed to improving the food security of people at risk or those in situations of social exclusion. 

The carbon footprint of the FBN was 147 tonnes of CO_2_e, of which 81% corresponded to fuel consumption in the transport of food and staff. Nevertheless, in a scenario without the FBN, the waste of food would be 32 times greater in terms of GHG emissions than in a scenario where the waste of food is avoided as per the FBN’s actions. In this hypothetical case without the activity of the FBN, the most significant impact would be represented by additional food production, which would account for 91% of the GHG emissions. In addition, the rest of the GHG emissions would be released during the management of unused food, according to the 2018 data. The results of the comparative analysis highlight the environmental benefits of the activity of the FBN, as it avoided the emission of 4568 and 4157 tonnes of CO_2_e in 2018 and 2019, respectively. The activities of food banks could therefore be included as projects suitable for the purposes of generating carbon offsets in the carbon offset markets, whether they be national, European, or global. This has hitherto not been accomplished and could incentivise not only a reduction in GHG emissions but also reductions in the impacts on other resources, such as water or biodiversity, as a parallel outcome of the reduction in food losses.

This kind of organisation leads to a very important model of a social and solidarity type of economy. Through its activities, it contributes to the three dimensions of sustainable development: social, economic, and environmental. Food banks are, therefore, key players in the development of a more sustainable food chain. Even if surplus donation alliances with food banks and other organisations are promoted by some national policies, such as in the first draft of the Spanish Food Waste Law, there is still a wide scope for improvement in terms of governmental policy orientation.

## Figures and Tables

**Table 1:** Percentage of carbon reduction per emission source if a carbon reduction plan was implemented by the Food Bank of Navarra, in comparison with the base year of 2018.

Recommendation	GHG Emission in 2018 (t CO_2_e)	GHG Emission after Applying Recommendations (t CO_2_e)	Avoided GHG Emission (t CO_2_e)	Carbon Footprint of the FBN 2018 (t CO_2_e)	Carbon Footprint of the FBN after the Recommendations (t CO_2_e)	Carbon Reduction (%)
Change to renewable electricity service at the FBN headquarters in Tudela	3	0	3	147	134	9
Increase the use of public transport (bus) by 25% for staff traveling by car to the FBN *	43	33	10			

* In 2018, 90% of the FBN volunteers travelled by car.

**Table 2:** Comparison of the results of the technical reports on the Food Bank of Cordoba [30], the Food Bank of Bordeaux [29], and the Food Bank of Navarra (present report).

	Impact Category	Food Bank of Navarra (2018) (t CO_2_ e)	Food Bank of Cordoba (t CO_2_ e)	Food Bank of Bordeaux (t CO_2_ e)
	Food distributed	2,767,536 kg	451,769 kg	4,500,000 kg
**Scenario with the food bank in** **T CO_2_ e**	Fixed combustion	9.6	11.2	NA
	Transportation fuel consumption	14.8		69
	Transport of food inflows	45.3		84.5
	Transport of food outflows	27.7		
	Transport of volunteers and staff	43.2		21.55
	Electricity consumption	3	5.6	1.9
	Refrigerant gas recharge	0	NA	NA
	Consumption of goods and services	3.6	NA	23.4
**Scenario without the food bank** **T CO_2_ eq**	Waste management	443	NA	102.9
	Additional food production	4272	564.6	8122
**Comparative analysis**	Scenario with the food bank Scenario without the food bank	4568	547.9	8025

NA = No Analised.

## Data Availability

Data is contained within the article.

## Supplementary Materials

The following supporting information can be downloaded at: https://www.mdpi.com/article/10.3390/foods11223645/s1, Figure S1: Comparison of greenhouse gas emissions from the activities of the Food Bank of Navarra by scope between 2018 and 2019 (tonnes CO_2_e); Figure S2: Comparison of greenhouse gas emissions by category in a scenario “without the action of the Food Bank of Navarra” between 2018 and 2019 (tonnes of CO_2_e); Figure S3: Comparison of total greenhouse gas emissions in the scenarios "with" and "without" the action of the Food Bank of Navarra, and emissions avoided by the Food Bank of Navarra between 2018 and 2019 (tonnes of CO_2_e); Table S1: Activities and emission factors of the Food Bank of Navarra carbon footprint, Scope 1 (2018); Table S2: Activities and emission factors of the Food Bank of Navarra carbon footprint, Scope 2 (2018); Table S3: Activities and emission factors of the Food Bank of Navarra carbon footprint, Scope 3, Upstream. Transport associated with food inputs (2018); Table S4: Activities and emission factors of the Food Bank of Navarra carbon footprint, Scope 3, Downstream. Transport to social entities (2018); Table S5: Activities and emission factors of the Food Bank of Navarra carbon footprint, Scope 3, Total transport of goods (2018); Table S6: Activities and emission factors of the Food Bank of Navarra carbon footprint, Scope 3. Downstream: Staff and volunteer transport, Transport of volunteers, private vehicle (2018); Table S7: Activities and emission factors of the Food Bank of Navarra carbon footprint, Scope 3. Downstream: Transport of volunteers. Public transport (2018); Table S8: Activities and emission factors of the Food Bank of Navarra carbon footprint, Scope 3. Downstream: Total volunteer transport (2018); Table S9: Activities and emission factors of the Food Bank of Navarra carbon footprint, Scope 3. Consumption of goods and services (2018); Table S10: Greenhouse gas emissions from the different activities of the Food Bank of Navarra by scope in 2018 (tonnes of CO_2_e); Table S11: Activities and emission factors in a scenario without the FBN, Additional food production (2018); Table S12: Activities and emission factors in a scenario without the FBN, Waste management, products whose donors are in the PRC (2018); Table S13: Activities and emission factors in a scenario without the FBN, Waste management, Products originating in Navarra (except for the PRC) (2018); Table S14: Activities and emission factors in a scenario without the FBN, Waste management, Products originating in the rest of Spain (except Catalonia) (2018); Table S15: Activities and emission factors in a scenario without the FBN, Waste management, Products originating in Catalonia (2018); Table S16: Activities and emission factors in a scenario without the FBN, total Waste management in the rest of Spain (2018); Table S17: Activities and emission factors in a scenario without the FBN, Waste management total data (2018). Table S18: Quantity (tonnes) and GHG emissions (in tonnes CO_2_e and percentage) of food donations by food category to the Food Bank of Navarra in 2018.

## Abbreviations

AD	Activity data
ADEME	French Agency for Ecological Transition
CO_2_e	CO_2_ equivalent
E	Emission of greenhouse gases
EF	Emission factor
FBN	Food Bank of Navarra
FVPO	Fruit and vegetable producer organisation
GHG	Greenhouse gas
PRC	Pamplona Region Commonwealth
SDG	Sustainable development goal
US	The United States of America
