# Supplementary material for: Food Banks against Climate Change, a Solution That Works: A Case Study in Navarra, Spain
Source: Foods. 2022 Nov 15;11(22):3645. doi: 10.3390/foods11223645 (PMC9689619; doi:10.3390/foods11223645)
Supplement: Supplementary file 1 [file foods-11-03645-s001.zip › foods-1916521-supplementary.pdf]

Supplementary material for

Food banks against climate change, a solution that works: the case of Navarra, Spain

Josemi G. Penalver [1], Alejandra Armijos [2], Beatriz Soret [3,4] and Maite M. Aldaya [4]*

[1] School of Agricultural Engineering and Biosciences, Public University of Navarra (UPNA), Arrosadia Campus, 31006 Pamplona, Spain
[2] Institute for Sustainability & Food Chain Innovation (IS-FOOD), Public University of Navarra (UPNA), Arrosadia Campus, 31006 Pamplona, Spain
* Correspondence: maite.aldaya@unavarra.es

Table S1: Activities and emission factors of the Food Bank of Navarra carbon footprint, Scope 1 (2018).

Emission source	Activity data	Unit	Emission factor	Unit	Comments	Source	Emissions	Unit
**Consumption of natural gas**	52442	kWh	0.183	kg CO_2/kWh	Year 2018	MITECO (2019)	9597	kg CO_2
**Vehicle type**	**Activity data**	**Unit**	**Fuel type**	**Emission factor**	**Unit**	**Source**	**Emissions**	**Unit**
**FBN´s van**	5951.5	litre	Diesel A or B	2.493	kg CO_2e/litre	MITECO (2020)	14837	kg CO_2
**Vehicle type**	**Activity data**	**Unit**	**Gas name**	**Emission factor**	**Unit**	**Source**	**Emissions**	**Unit**
**Recharging of refrigerant gases**	0	litre			kg CO_2e/litre	MITECO (2020)	0	kg CO_2

MITECO, Ministerio para la Transición Ecológica y el Reto Demográfico (2019 and 2020) *Carbon footprint calculator* [Online] https://www.miteco.gob.es/es/cambio-climatico/temas/mitigacion-politicas-y-medidas/calculadoras.aspx.

Table S2: Activities and emission factors of the Food Bank of Navarra carbon footprint, Scope 2 (2018).

Facilities	Emission source	Activity data	Unit	Trading company	Emission factor	Unit	Comments	Source	Emissions	Unit

**Pamplona**	Electricity consumption	74023	kWh	EMASP S. COOP	0	Kg CO_2e/kWh	Year 2018, wind energy	MITECO (2019)	0	kg CO_2e
**Tudela**	Electricity consumption	10185	kWh	Iberdrola Clientes S.A.U.	0.27	Kg CO2e/kWh	Year 2018	MITECO (2019)	2750	kg CO_2e
**Total**									**2750**	**kg CO_2e**

MITECO, Ministerio para la Transición Ecológica y el Reto Demográfico (2019) *Carbon footprint calculator* [Online] https://www.miteco.gob.es/es/cambio-climatico/temas/mitigacion-politicas-y-medidas/calculadoras.aspx.

Table S3: Activities and emission factors of the Food Bank of Navarra carbon footprint, Scope 3, Upstream. Transport associated with food inputs (2018).

**Origin**	**Type of vehicle**	**Fuel type****	**Distance travelled**	**Unit**	**Speed**	**Emission factor**	**Unit**	**Source**	**Emissions**	**Unit**
**Manufacturers and distributors ***	Van (19%)	Gasoline (17%)	331	km	High (87 km/h)	0.26	kg CO_2e/km	OCCC (2020)	87	kg CO_2e
		Gasoil (83%)	1614	km	High (87 km/h)	0.20	kg CO_2e/km	OCCC (2020)	330	kg CO_2e
	Rigid lorry > 14 t (31%)	-	3173	km	High (87 km/h)	0.45	kg CO_2e/km	OCCC (2020)	1436	kg CO_2e
	Articulated lorry <= 34 t (47%)	-	4811	km	High (87 km/h)	0.50	kg CO_2e/km	OCCC (2020)	2406	kg CO_2e
	Articulated lorry > 34 t (3%)	-	307	km	High (87 km/h)	0.56	kg CO_2e/km	OCCC (2020)	172	kg CO_2e
	**Total**	-	**10236**	**km**					**4431**	**kg CO_2e**
**The Fund for European Aid to the Most Deprived program***	Articulated lorry > 34 t	-	23350	km	High (87 km/h)	0.56	kg CO_2e/km	OCCC (2020)	13075	kg CO_2e
**Food collections***	Rigid lorry < 14 t (5%)	-	213	km	High (87 km/h)	0.40	kg CO_2e/km	OCCC (2020)	85	kg CO_2e

	Rigid lorry > 14 t (10%)	-	426	km	High (87 km/h)	0.45	kg CO_2e/km	OCCC (2020)	193	kg CO_2e
	Articulated lorry <= 34 t (36%)	-	1464	km	High (87 km/h)	0.50	kg CO_2e/km	OCCC (2020)	732	kg CO_2e
	Articulated lorry > 34 t (49%)	-	2011	km	High (87 km/h)	0.56	kg CO_2e/km	OCCC (2020)	1126	kg CO_2e
	**Total**		**4114**	**km**					**2136**	**kg CO_2e**
**PRC**	Van	Gasoline (15.4%)	8609	km	Average between urban speed(21 km/h) and average speed (62 km/h)	0.26	kg CO_2e/km	OCCC (2020)	2271	kg CO_2e
		Gasoil (84.6%)	47296	km	Average between urban speed(21 km/h) and average speed (62 km/h)	0.20	kg CO_2e/km	OCCC (2020)	9681	kg CO_2e
	**Total**	-	**55905**	**km**					**11951**	**kg CO_2e**
**Fruit and Vegetable Producers Organisation**	Articulated lorry > 34 t	-	23397	km	High (87 km/h)	0.56	kg CO_2e/km	OCCC (2020)	13101	kg CO_2e
**Others food banks**	Articulated lorry<=34t	-	1110	km	High (87 km/h)	0.50	kg CO_2e/km	OCCC (2020)	555	kg CO_2e
**Food donations**	Passenger car (75%)	Gasoline (38.0%)	48	km	Average between urban speed(21	0.185	kg CO_2e/km	OCCC (2020)	9	kg CO_2e

				km/h) and average speed (69 km/h)					
	Gasoil (62.0%)	78	km	Average between urban speed(21 km/h) and average speed (69 km/h)	0.166	kg CO_2e/km	OCCC (2020)	13	kg CO_2e
Van (25%)	Gasoline (15.4%)	6	km	Average between urban speed(21 km/h) and average speed (62 km/h)	0.26	kg CO_2e/km	OCCC (2020)	2	kg CO_2e
	Gasoil (84.6%)	36	km	Average between urban speed(21 km/h) and average speed (62 km/h)	0.20	kg CO_2e/km	OCCC (2020)	7	kg CO_2e
**Total**		168						**31**	**kg CO_2e**
**Total transport associated with food inflows**								**45281**	kg CO_2e

OCCC, Oficina Catalana del Cambio Climático (2020) *Calculation of GHG emissions derived from municipal waste management.*

* Part of the transport carried out by the FBN.

** Percentages according to the number of vehicles in Navarre in the case of the PRC and collections. Percentage according to the number of vehicles in Spain in the case of Manufacturers and Distributors.

Table S4: Activities and emission factors of the Food Bank of Navarra carbon footprint, Scope 3, Downstream. Transport to social entities (2018).

Destination	Type of vehicle	Fuel type*	Distance travelled	Unit	Speed	Emission factor	Unit	Source	Emissions	Unit
**Entities in Navarre**	Passenger car	Gasoline 38.0%	24817	km	High (102 km/h)	0.15	kg CO_2e/km	OCCC (2020)	3723	kg CO_2e
	Passenger car	Gasoil 62.0%	40491	km	High (102 km/h)	0.15	kg CO_2e/km	OCCC (2020)	5899	kg CO_2e
	Van	Gasoline 15.4%	10057	km	High (92 km/h)	0.19	kg CO_2e/km	OCCC (2020)	1872	kg CO_2e
	Van	Gasoil 84.6%	55250	km	High (92 km/h)	0.18	kg CO_2e/km	OCCC (2020)	10130	kg CO_2e
	**Total**		**130616**	**km**					**21624**	**kg CO_2e**
**Others food banks**	Articulated lorry<=34t	-	11677	km	High (87 km/h)	0.50	kg CO_2e/km	OCCC (2020)	5840	kg CO_2e
**Total transport to social entities**									**27463**	**kg CO_2e**

OCCC, Oficina Catalana del Cambio Climático (2020) *Calculation of GHG emissions derived from municipal waste management.*

* Fuel type according to the percentage of the vehicle fleet in Navarre. Year 2018.

Table S5: Activities and emission factors of the Food Bank of Navarra carbon footprint, Scope 3, Total transport of goods (2018).

	Emissions	Unit
**Total transport of food**	**72744**	**kg CO_2e**

Table S6: Activities and emission factors of the Food Bank of Navarra carbon footprint, Scope 3. Downstream: Staff and volunteer transport, Transport of volunteers, private vehicle (2018).

Origin	Type of vehicle	Fuel type	Distance travelled (km)	Speed	Emission factor kg CO_2e/km	Source	Emissions Kg CO_2e
**Pamplona**	Motorbike	Gasoline	5670	Average between urban speed(25 km/h) and average speed (69 km/h)	0.099	OCCC (2020)	562
**Pamplona**	Passenger car	Diesel	130617	Average between urban speed(21 km/h) and average speed (69 km/h)	0.166	OCCC (2020)	21737
	Passenger car	Gasoline	96205	Average between urban speed(21 km/h) and average speed (69 km/h)	0.185	OCCC (2020)	17776
	Passenger car	Hybrid	8910	Average between urban speed(21 km/h) and average speed (69 km/h)	0.093	OCCC (2020)	826
**Pamplona**	Van	Diesel	4320	Average between urban speed(21 km/h) and average speed (62 km/h)	0.205	OCCC (2020)	884
**Tudela**	Passenger car	Diesel	2880	Average between urban speed(21 km/h) and average speed (69 km/h)	0.166	OCCC (2020)	479
**Tudela**	Passenger car	Gasoline	6165	Average between urban speed(21 km/h) and average speed (69 km/h)	0.185	OCCC (2020)	1139
**Total**							**43404**

OCCC, Oficina Catalana del Cambio Climático (2020) *Calculation of GHG emissions derived from municipal waste management.*

Table S7: Activities and emission factors of the Food Bank of Navarra carbon footprint, Scope 3. Downstream: Transport of volunteers. Public transport (2018).

Origin	Distance travelled (km)	Average occupancy*	Unit	Emission factor	Unit	Source	Emissions Kg CO_2e
**City bus Pamplona**	10560	16	Passengers/bus	0.08074	kg CO_2e/Passenger/km	OCCC (2020)	53

OCCC, Oficina Catalana del Cambio Climático (2020) *Calculation of GHG emissions derived from municipal waste management.*

*Average occupation according to OCCC.

Table S8: Activities and emission factors of the Food Bank of Navarra carbon footprint, Scope 3. Downstream: Total volunteer transport (2018).

	Emissions	Unit
**Total staff transport**	**43457**	**kg CO_2e**

Table S9: Activities and emission factors of the Food Bank of Navarra carbon footprint, Scope 3. Consumption of goods and services (2018).

Emission source	Activity data	Unit	Emission factor	Unit	Comments	Source	Others	Emissions (Kg CO_2e)	Uncertainty
**Purchase of cardboard ***	8.85	T of cardboard	390	kg CO_2e/t of cardboard	Weight of one box (200 gr)	ADEME	New cardboard (not recycled)	3452	20%
**Purchase of wood ***	0.82	T of wood	36.7	kg CO_2e/t of wood	Weight of one box (2 kg)	ADEME	Short-lived wood (furniture ...) - upstream manufacture	30	50%
**Drinking water supply 1**	140	m^3	0.081	kg CO_2e/m^3		PRC (2018)		11	
**Drinking water supply 2**	126	m^3	0.081	kg CO_2e/m^3		PRC (2018)		10	
**Total**								**3503**	

ADEME, Agence de la transition écologique (2020) Base de données Bilan Carbone, Base Carbone V11 [Online] https://www.bilans-ges.ademe.fr/en/accueil.

PRC (2018) personal communication from Álvaro Miranda, from the PRC, April 16, 2020

* These purchases are of industrial packaging for the transport and storage of foodstuffs.

Table S10: Greenhouse gas emissions from the different activities of the Food Bank of Navarra by scope in 2018 (tonnes of CO_2e)

		**Emissions (t CO_2e)**	**Emissions (%)**
**Scope 1**	Fixed combustion	9.6	6.5 %
	Transportation fuel consumption	14.8	10.1 %
	refrigerant gas recharge	0	0 %
**Scope 2**	Electricity consumption in Berrioplano	0	0 %
	Electricity consumption in Tudela	3	2 %
**Scope 3**	Transport of food inflows	45.3	30.8 %
	Transport of food outflows	27.7	18.8 %
	Transport of volunteers and staff	43.2	29.3 %
	Consumption of goods and services	3.6	2.4 %

Table S11: Activities and emission factors in a scenario without the FBN, Additional food production (2018).

**Category***	**Activity data**	**Units**	**Emission factor (kg CO_2e/kg o L)**	**Source**	**Others**	**Emissions (kg CO_2e)**	**Uncertainty**
**Infant food**							
**Infant food**	160	kg	2.00	Veolia (2016)	Baby food	321	
**Infant cereals in porridge**	118	kg	2.000	Veolia (2016)	Baby food	235	
**Unscheduled food**							
**Unscheduled food**	146540	kg	2.000	Veolia (2016)	Miscellaneous foodstuffs	293080	

**Frozen unsorted food**	68849	kg	3.475	Veolia (2016)	Average of fruits (0.671), vegetables (0.671), pasta (2.766), fish (2.870) and frozen meats (10.395).	239222	
**Drinks**							
**Flavoured shakes**	1409	l	1.3	ECODES MITECO (2019)	Shakes	1831	
**Water without gas**	15	kg	0.393	ADEME	PET bottled water, 0.5L - in the shop, ready to drink	6	30%
**Cola drink**	135	l	1.090	ADEME	Soda - cola - in shop, ready to drink	147	30%
**Orange soft drinks**	35	l	2.220	ADEME	Orange juice	78	30%
**Drink without gas**	422	l	1.307	ADEME	Average between PET water bottle and orange juice in shop	552	30%
**Assorted juices and nectars**	109573	kg	2.220	ADEME	Orange juice - pure juice - in shop, ready to drink	243252	30%
**A variety of soft drinks**	13899	kg	1.655	ADEME	Average between orange juice and cola soda	23002	30%
**Pastries, biscuits and sweets**							
**Assorted biscuits**	13131	kg	2.508	Veolia (2016)	Biscuits	32934	
**Sweet pastries**	379	kg	2.525	ADEME	Average cheesecake (0.764 $kgCO_2e$/serving, portion 0.217), strawberry tart (0.345 $kgCO_2e$/serving, portion 0.139) apple tart (0.283 $kgCO_2e$/serving, portion 0.18)	957	30%
**Assorted jams**	48	kg	2.176	Veolia (2016)	Confectionery	104	
**Assorted marmalades**	48866	kg	2.176	Veolia (2016)	Confectionery	106333	

**Assorted honey**	216	kg	0.960	Observatorio CO2web	Honey	208	
**Candies**	0	kg	2.176	Veolia (2016)		0	
**Confectionery**	134	kg	2.525	ADEME	Average cheesecake (0.764 $kgCO_2e$/serving, portion 0.217), strawberry tart (0.345 $kgCO_2e$/serving, portion 0.139) apple tart (0.283 $kgCO_2e$/serving, portion 0.18)	339	30%
**Nougats, polvorones and chocolates**	559	kg	2.176	Veolia (2016)	Confectionery	1216	
**Assorted pastries**	38214	kg	2.28	ADEME	pastries - chocolate pastry - net weight in shop	87128	30%
**Cocoa and chocolate**							
**Soluble cocoa (Colacao)**	2474	kg	4.700	ADEME	Chocolate powder, unsweetened - net weight in shop. Same perimeter as ingredients purchased in bulk. FE variation for this ingredient: Total product consumption - Conversion factor: 100%.	11629	30%
**Chocolate powder**	3	kg	4.700	ADEME	Chocolate powder, unsweetened - net weight in shop. Same perimeter as ingredients purchased in bulk. FE variation for this ingredient: Total product consumption - Conversion factor: 100%.	14	30%
**Chocolate sweets**	177	kg	5.870	ADEME	Milk chocolate, lumps - net weight in shop	1038	30%
**Chocolates in bars**	257	kg	5.870	ADEME	Milk chocolate, lumps - net weight in shop	1508	30%

**Coffee and infusions**							
**Assorted coffee**	488	kg	3.140	ADEME	Ground coffee - net weight in shop	1531	30%
**Assorted infusions**	1257	kg	6.110	ADEME	Tea - for infusion - net weight in shop	7679	30%
**Meats**							
**Cured ham**	425	kg	9.310	ECODES MITECO (2019)	Ham and cured pork shoulder	3959	
**Cured sausages, chorizo**	19691	kg	5.120	ADEME	Salchichon (pork)	100820	30%
**Assorted canned pâté**	6	kg	10.395	Veolia (2016)	Canned pâté	58	
**Packaged meats**	2	kg	4.390	ADEME	Pork sausage, but net in shop	11	30%
**Canned meat**	3	kg	4.39	ADEME	Pork sausage	11	30%
**Assorted cured sausages**	13379	kg	5.120	ADEME	Salchichon (pork)	68502	30%
**Assorted cold meats**	163	kg	5.120	ADEME	Salchichon (pork)	835	30%
**Cereals, flour, bread and pasta**							
**Various pastas**	1903	kg	1.48	ADEME	Paste - dry - net weight shop	2817	30%
**Macaroni**	2409	kg	1.48	ADEME	Paste - dry - net weight shop	3566	30%
**Spaguetti**	4624	kg	1.48	ADEME	Paste - dry - net weight shop	6844	30%
**Rice**	14709	kg	4.230	ADEME	Rice - jasmine, Thailand - net weight in shop	62219	30%
**Corn**	9	kg	0.869	ADEME	Cereal bowl - ready-to-eat dishes. Mass of consumable ingredients in the bowl for one serving (kg): 0.337	8	30%

**Sliced bread**	10305	kg	1.520	ADEME	Bread - net weight in shop. Wheat flour	15664	30%
**Toasted bread**	5067	kg	1.520	ADEME	Bread - net weight in shop. Wheat flour	7702	30%
**Breakfast cereals**	1824	kg	0.869	ADEME	Cereal bowl - ready-to-eat dishes. Mass of consumable ingredients in the bowl for one serving (kg): 0.337	1586	30%
**Flour**	1774	kg	1.170	ADEME	Flour - wheat - net weight in shop	2075	30%
**Condiments and sauces**							
**Assorted vinegar**	573	l	4.17	ADEME	Wine vinegar - net weight in shop. 1.2 kg of grapes to make 1 litre of wine vinegar.	2391	30%
**Salt**	2	kg	0.544	ADEME	Salt - net weight in shop	1	30%
**Oil**	9995	kg	2.11	ADEME	Half olive and sunflower oil - net weight in shop.	21089	30%
**Sugar**	2385	kg	0.682	ADEME	Beet sugar, refined - net weight in shop. 90% of sugar consumption in France	1626	30%
**Assorted prepared sauces**	30336	kg	2.940	ADEME	Tomato sauce - net weight in shop. Same perimeter as ingredients purchased in bulk.	89188	30%
**Mayonnaise**	2128	kg	2.000	Veolia (2016)	Miscellaneous foods	4255	
**Ketchup**	16347	kg	2.4	ADEME	Ketchup - net weight in shop	39232	30%
**Mustard**	3565	kg	3.27	ADEME	Condiment. Mustard - net weight in shop	11658	30%
**Canned vegetables/legumes**							

**Cooked red kidney beans**	17658	kg	0.873	Veolia (2016)	Canned vegetables/legumes	15415
**Cooked or canned mixed legumes**	86097	kg	0.873	Veolia (2016)	Canned vegetables/legumes	75163
**Cooked beans with vegetables**	14873	kg	0.873	Veolia (2016)	Canned vegetables/legumes	12985
**Cooked chickpeas**	20427	kg	0.873	Veolia (2016)	Canned vegetables/legumes	17833
**Cooked lentils in their natural state**	95107	kg	0.873	Veolia (2016)	Canned vegetables/vegetables	83028
**Canned vegetables artichoke hearts**	3432	kg	0.873	Veolia (2016)	Canned vegetables	2996
**Canned vegetables white asparagus**	5089	kg	0.873	Veolia (2016)	Canned asparagus, Origin China, net weight in shop	4443
**Canned vegetables thin peas**	27739	kg	0.873	Veolia (2016)	Canned peas, net weight in shop	24216
**Canned vegetables green beans**	9754	kg	0.873	Veolia (2016)	Canned vegetables	8515
**Canned vegetables maíz dulce**	8785	kg	0.873	Veolia (2016)	Canned vegetables	7669
**Canned vegetables cooked chard**	11487	kg	0.873	Veolia (2016)	Canned vegetables	10028
**Canned vegetables celery strips**	5904	kg	0.873	Veolia (2016)	Canned vegetables	5154
**Canned vegetables mixed vegetables**	325	kg	0.873	Veolia (2016)	Canned vegetables	284
**Canned vegetables whole potatoes**	5437	kg	0.873	Veolia (2016)	Canned vegetables	4747
**Canned vegetables cooked beetroot**	261	kg	0.873	Veolia (2016)	Canned vegetables	228
**Canned vegetables canned peppers piquillo peppers**	1504	kg	0.873	Veolia (2016)	Canned vegetables	1313

**Canned vegetables mushroom**	866	kg	0.873	Veolia (2016)	Canned vegetables	756	
**Canned vegetables carrot**	1193	kg	0.873	Veolia (2016)	Canned vegetables	1042	
**Canned mixed vegetables**	47737	kg	0.873	Veolia (2016)	Canned vegetables	41675	
**Fruits**							
**Cherry**	13824	kg	0.663	ADEME	Cherry - net weight in shop	9165	30%
**Plum**	26000	kg	0.259	ADEME	Fruit (or vegetable) - generic, seasonal, locally produced - net in-store weight	6734	30%
**Apple**	30890	kg	0.259	ADEME	Apple France - net weight in shop	8000	30%
**Peach**	19863	kg	0.346	ADEME	Peach France - net weight in shop	6873	30%
**Melon**	23360	kg	0.313	ADEME	Melon - net weight in shop	7312	30%
**Orange**	140940	kg	0.500	ADEME	Half orange, fresh and for industry - net weight in shop	70470	30%
**Nectarine**	22530	kg	0.346	ADEME	Peach France - net weight in shop	7795	30%
**Pear**	38151	kg	0.45	ADEME	Representative of a pear produced in Belgium, use this value for French production.	17168	30%
**Papaya**	44552	kg	2.24	ADEME	Fruit (or vegetable) - generic, out of season, produced in a climate-controlled greenhouse - net in-store weight	99796	30%
**Banana**	11618	kg	0.698	ADEME	Banana - net retail weight	8109	30%
**Grape**	6525	kg	0.642	ADEME	Grapes - net weight in shop	4189	30%
**Fresh fruit**	57050	kg	0.259	ADEME	Fruit (or vegetable) - generic, seasonal, locally produced - net weight in shop	14776	30%
**Tangerines**	267574	kg	0.767	ADEME	Tangerine - net weight in shop	205230	30%

**Canned fruit**							
**Canned fruit, assorted**	939	kg	2.176	Veolia (2016)	Canned Fruit	2044	
**Peach in syrup**	5678	kg	2.176	Veolia (2016)	Canned Fruit	12356	
**Pineapple in juice**	43	kg	2.176	Veolia (2016)	Canned Fruit	93	
**Nuts**							
**Nuts**	318	kg	0.663	ADEME	Walnuts	211	30%
**Walnuts**	131	kg	0.663	ADEME	Whole walnuts - net weight in shop	87	30%
**Vegetables and pulses**							
**Garlic**	3000	kg	0.57	Observatorio CO2web	Place of calculation: United Kingdom	1710	
**Pumpkin**	10792	kg	0.729	ADEME	Pumpkin - net shop weight	7867	30%
**Onion**	35610	kg	0.485	ADEME	Onions - net shop weight	17271	30%
**Lettuce**	25440	kg	0.479	ADEME	Salad - net shop weight - Agricultural production system: espYearla	12186	30%
**Sweet corn**	544	kg	0.701	ADEME	Maize - net shop weight	381	30%
**Potatoes**	76707	kg	0.585	ADEME	Potato - net shop weight	44874	30%
**Cucumbers**	22699	kg	1.38	ADEME	Cucumber - seasonal - net weight in shop	31324	30%
**Peppers**	2728	kg	0.871	ADEME	Pepper - net shop weight	2376	30%
**Tomatoes**	94872	kg	0.343	ADEME	Tomato - fresh seasonal, France - net in-store weight	32541	30%

**Fresh vegetables**	91024	kg	0.259	ADEME	Fruit (or vegetable) - generic, seasonal, locally produced - net weight in shop	23575	30%
**Tomato preserves**	242519	kg	1.41	ADEME	Tomato - pulp or peeled - net weight in shop	341951	30%
**Lentils**	149	kg	0.887	ADEME	Green lentils - net weight in shop	132	30%
**Various pulses**	1610	kg	0.887	ADEME	Green lentils - net weight in shop	1428	30%
**Eggs**							
**Eggs**	6596	kg	2.090	ADEME	Egg - national average - net weight in shop. Representing the French egg market, with an industrial part and an outdoor/label part.	13785	30%
**Quail eggs**	23	kg	2.090	ADEME	Egg - national average - net weight in shop. To represent the French egg market, with an industrial part and an outside/label part.	48	30%
**Fish**							
**Tuna in vegetable oil**	39	kg	3.160	ADEME	Canned tuna - net weight in shop	124	30%
**Canned fish, assorted**	781	kg	3.70	Veolia (2016)	Canned fish. Exotic fish and canned tuna	2890	
**Ready to eat meals**							
**Assorted frozen ready to eat meals**	6908	kg	6.780	Veolia (2016)	Prepared meals. Based on the ADEME V11 "composite meal"	46836	30%
**Canned ready to eat meals assorted**	681	kg	4.389	ADEME	Average of the following ready meals: lasagne (2.91 $kgCO_2e$/serving, serving 0.594), beans with meat (2.37	2989	30%

					kgCO_2e/serving, serving 0.37), pasta with ham (1.36 kgCO_2e/serving, serving 0.535), chicken with curry rice (1.19 kgCO_2e/serving, serving 0.567), cou cous (4.92 kgCO_2e/serving, serving 0.82).		
**Assorted ready to eat meals**	50219	kg	6.78	Veolia (2016)	Prepared meals. Based on the ADEME V11 "composite meals"	340484	
**Frozen pizzas**	805	kg	5.112	ADEME	Pizza: ready-to-eat meals. Mass of consumable ingredients for one portion (kg): 0.143	4114	30%
**Chilled pizzas and Others**	3302	kg	5.112	ADEME	Pizza: ready-to-eat dishes. Mass of consumable ingredients for one portion (kg): 0,143	16881	30%
**Assorted soups**	60	kg	0.873	Veolia (2016)	Soups	52	
**Various broths**	29144	kg	0.873	Veolia (2016)	Soups	25443	
**Gazpacho**	72	kg	2.000	Veolia (2016)	Miscellaneous foods	144	
**Assorted creams**	11189	kg	5.320	Observatorio CO2web	Creams	59523	
**Hygiene products**							
**Grooming drugstore**	1319	kg	2.000	Veolia (2016)	Hygiene products	2638	
**Cleaning drugstore**	80	kg	2.000	ADEME		159	30%
**Diapers**	1034	kg	2.000	Veolia (2016)	Hygiene products	2068	
**Dairy products**							
**Assorted dairy desserts**	9591	kg	2.52	ECODES MITECO (2019)	dairy desserts	24170	

**assorted yogurts**	253661	kg	2.88	ADEME	Yogurt - store net weight. Quantity of milk considered for a kg of yogurt: 1.5 kg. Approximation to use any type of yogurt (flavored, whole milk, etc.)	730545	30%
**Custard**	579	kg	2.90	ADEME	Créme brûlée - ready-to-eat dishes. One serving: 0.187	1681	30%
**fresh cheeses**	446	kg	3.510	ADEME	Fresh cow cheese, 58% fat - net store weight	1567	30%
**assorted cheeses**	713	kg	4.94	ADEME	Average of hard cheese (Emmental type) and soft cheese (Camembert type) - net weight in store	3524	30%
**assorted milk**	89925	l	1.22	ADEME	Milk - cow's milk, semi-skimmed, pasteurized - store net weight. Approximation to use in all milks	109709	30%
**fresh milk**	1130	kg	1.220	ADEME	Milk - cow's milk, semi-skimmed, pasteurized - store net weight. Approximation to use in all milks	1379	30%
**Milk powder**	80	kg	2.000	Veolia (2016)	various foods	160	
**butters**	2428	kg	9.490	ADEME	Sweet butter - net store weight	23042	30%
**Varied snacks**							
**Chips**	1622	kg	1.776	ADEME	chips. Portion 0.196	2879	30%
**Fries and assorted snacks**	9646	kg	1.776	ADEME	chips. Portion 0.196	17127	30%
**Assorted cereal bar**	7792	kg	2.000	Veolia (2016)	various foods	15585	
**assorted olives**	7331	kg	0.858	ADEME	Olives in jar, net weight in store	6290	30%
**Total**	2767536	kg				4271808	

ADEME, Agence de la transition écologique (2020) Base de données Bilan Carbone, Base Carbone V11 [Online] https://www.bilans-ges.ademe.fr/en/accueil.

Cátedra de Ética Ambiental (2020) Observatorio CO2 web [Online] Fundación Tatiana Pérez de Guzmán el Bueno, Universidad de Alcalá. https://huellaco2.org/alimentos.php.

ECODES, Fundación Ecología y Desarrollo (2019) *Prevención de la contaminación e impacto climático en función de la selección de las diferentes alternativas de alimentación. Fundación Ecología y Desarrollo.* Ministerio para la Transición Ecológica de España. Retrieved from https://ecodes.org/documentos/4_Documentacion-MITECO.pdf

Guilhem Julien (2016) *Rapport sur l'empreinte environnementale liee a l'activite de la banque alimentaire de bordeaux & de la gironde en 2015.* Veolia Environnement S.A. for the the food bank of bordeaux & gironde. Bordeaux, France. Technical report.

* The products considered are those that avoid food waste:

- Manufacturers and distributors

- Fruits and vegetables from market withdrawals (the Fruit and Vegetable Producers Organisation)

- PRC (surplus from commercial surfaces)

- Other Food Banks

Table S12: Activities and emission factors in a scenario without the FBN, Waste management, products whose donors are in the PRC (2018).

Origin	Waste fractions*	Unit	Activity data (kg)	Emission factor ($kgCO_2eq/kg$)	Source	Comments	Emissions kg CO_2e
**Landfill**	9.1	%	70829	0.624	PRC (2018)	0.624 tCO_2eq/ t of organic matter during 30 Years	44198
**Organic matter**	49.0	%	381357				34785
**Biomethanisation**	53.0	%	202119	0.020	MITECO (2020)	Spain	4042
**Composting**	47.0	%	179238	0.172	MITECO (2020)	Spain	30743
**Paper and cardboard**	8.7	%	67375	0.052	OCCC (2020)	Catalonia	3512
**Light packaging**	27.2	%	211299	0.016	OCCC (2020)	Catalonia	3429
**Glass**	6.1	%	47277	0.007	OCCC (2020)	Catalonia	354
**Total**	**100**	**%**	**778137**				**86278**

OCCC, Oficina Catalana del Cambio Climático (2020) *Calculation of GHG emissions derived from municipal waste management.*

Gobierno de Navarra (2020) *Oficina de Prevención de Residuos y de Impulso de la Economía Circular, Limitaciones que Abren Puertas* [Online] [Cited: 4 may 2022] https://oprec-navarra.com/limitaciones-abren-puerta/.

MITECO, Ministerio para la Transición Ecológica y el Reto Demográfico (2020) *Inventario Nacional de Gases de Efecto Invernadero.* Gobierno de España.

PRC (2018) personal communication from Álvaro Miranda, from the PRC, April 16, 2020.

* Inventory of household and commercial waste for the PRC (Government of Navarra, 2019) reweighted to exclude the "Other" fraction composed of items not handled by the FBN (batteries, medicines, electrical and electronic products). Values reweighted according to the composition percentages of the waste fraction container.

Table S13: Activities and emission factors in a scenario without the FBN, Waste management, Products originating in Navarra (except for the PRC) (2018).

Origin	Waste fractions (%)	Activity data (kg)	Emission factor	Unit	Source	Comments	Emissions	Unit
**Landfill**	35.0	298601	0.625	kgCO_2eq/kg	OCCC (2020)	Catalonia	186479	kg CO_2e
**Biomethanisation**	64.1	547213	0.020	kgCO_2eq/kg	MITECO (2020)	Spain	10944	kg CO_2e
**Light packaging**	0.1	740	0.016	kgCO2eq/kg	OCCC (2020)	Catalonia	12	kg CO_2e
**Refuse**	0.8	6590	-	-				
**Total**	**99**	**853145**					**197435**	**kg CO_2e**

OCCC, Oficina Catalana del Cambio Climático (2020) *Calculation of GHG emissions derived from municipal waste management.*

MITECO, Ministerio para la Transición Ecológica y el Reto Demográfico (2020) *Inventario Nacional de Gases de Efecto Invernadero.* Gobierno de España.

Table S14: Activities and emission factors in a scenario without the FBN, Waste management, Products originating in the rest of Spain (except Catalonia) (2018).

C.1. Rest of Spain	Waste fractions * (%)	Activity data** (kg)	Emission factor	Unit	Source	Comments	Emissions	Unit
**Landfill**	35.0	198845	0.625	$kgCO_2eq/kg$	OCCC (2020)	Catalonia	124181	kg CO_2e
**Biomethanisation**	64.1	364403	0.020	$kgCO_2eq/kg$	MITECO (2020)	Spain	7288	kg CO_2e
**Light packaging**	0.1	493	0.016	$kgCO_2eq/kg$	OCCC (2020)	Catalonia	8	kg CO_2e
**Refuse**	0.8	4389	-	-				
**Total**	**100**	**568130**					**131477**	**kg CO_2e**

OCCC, Oficina Catalana del Cambio Climático (2020) *Calculation of GHG emissions derived from municipal waste management.*

MITECO, Ministerio para la Transición Ecológica y el Reto Demográfico (2020) *Inventario Nacional de Gases de Efecto Invernadero.* Gobierno de España.

* Waste fractions taken from the waste managers of the FBN in Navarra.

**Data on the activity of distributors and manufacturers, other Food Banks and the Fruit and Vegetable Producers Organisation.

Table S15: Activities and emission factors in a scenario without the FBN, Waste management, Products originating in Catalonia (2018).

C.2. Rest of Spain	Waste fractions * (%)	Activity data** (kg)	Emission factor	Unit	Source	Comments	Emissions	Unit
**Landfill**	5.8	33208	0.625	$kgCO_2eq/kg$	OCCC (2020)	Catalonia	20739	kg CO_2e
**Paper and cardboard**	19.8	112419	0.052	$kgCO_2eq/kg$	OCCC (2020)	Catalonia	5860	kg CO_2e
**Light packaging**	16.6	94395	0.016	$kgCO_2eq/kg$	OCCC (2020)	Catalonia	1532	kg CO_2e
**Others*** **	57.8	328107	-	-				
**Total**	**100**	**568130**					**28131**	**kg CO_2e**

OCCC, Oficina Catalana del Cambio Climático (2020) *Calculation of GHG emissions derived from municipal waste management.*

* Waste fractions taken from FBN's waste managers in Catalonia.

**Data on the activity of distributors and manufacturers, other Food Banks and the Fruit and Vegetable Producers Organisation.

***Other refers to: specific treatments, other, management through a collection and transfer centre, use in agriculture, municipal collection, recycling and reuse of wood.

Table S16: Activities and emission factors in a scenario without the FBN, total Waste management in the rest of Spain (2018).

Total rest of Spain	Emissions	Unit
**Total**	**159608**	**kg CO_2e**

Table S17: Activities and emission factors in a scenario without the FBN, Waste management total data (2018).

Total waste management	Emissions	Unit
**Total**	**443322**	**kg CO_2e**

Table S18: Quantity (tonnes) and GHG emissions (in tonnes CO_2e and percentage) of food donations by food category to the Food Bank of Navarra in 2018.

Food Category	Quantity (t)	GHG Emissions (kg CO_2e/kg)	GHG Emissions (%)
**Dairy products**	359	896	21.0
**Unscheduled food**	215	532	12.5
**Vegetables and legumes**	608	518	12.1
**Prepared dishes**	102	496	11.6
**Fruits**	703	466	10.9
**Canned vegetables/legumes**	364	317	7.4
**Beverages**	125	269	6.3
**Pastries, biscuits and sweets**	102	229	5.4
**Meats**	34	174	4.1

**Condiments and sauces**	65	169	4.0
**Cereals, flour, bread and pasta**	43	102	2.4
**Assorted snacks**	26	42	1.0
**Preserved fruits**	7	14	0.3
**Cocoa and chocolate**	3	14	0.3
**Eggs**	7	14	0.3
**Coffees and infusions**	2	9	0.2
**Fish**	1	3	0.1
**Children's food**	0	1	0.01
**Nuts**	0	0	0.001
**Hygiene products**	2	5	0.1
**Total**	**2768**	**4272**	**100.0**

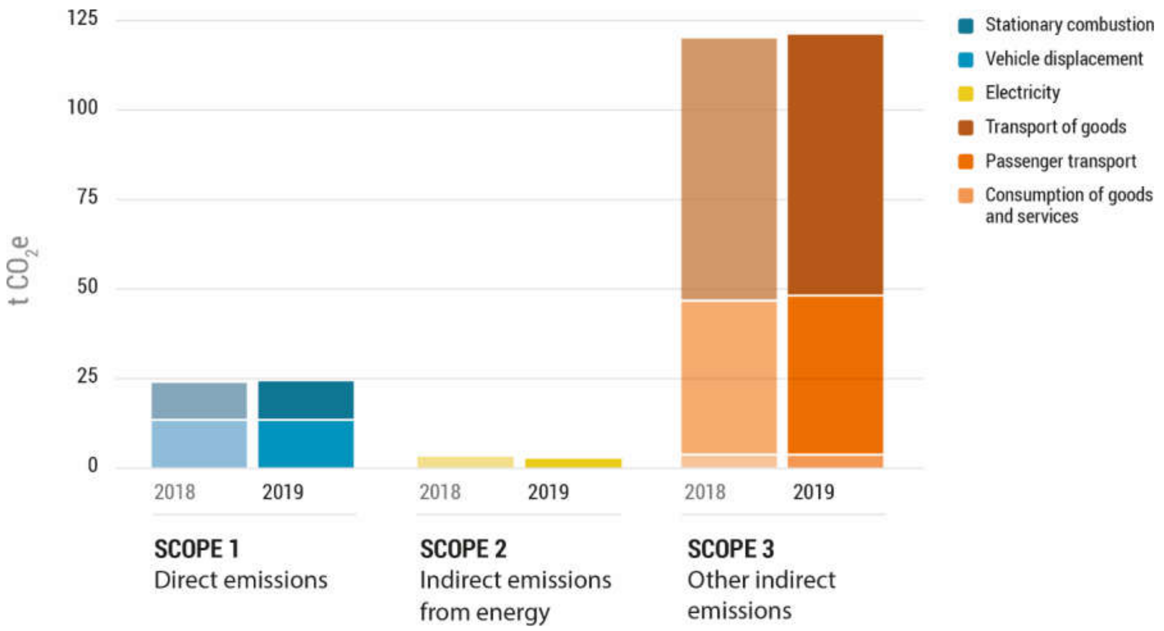

Figure S1: Comparison of greenhouse gas emissions from the activities of the Food Bank of Navarra by scope between 2018 and 2019 (tonnes CO_2e).

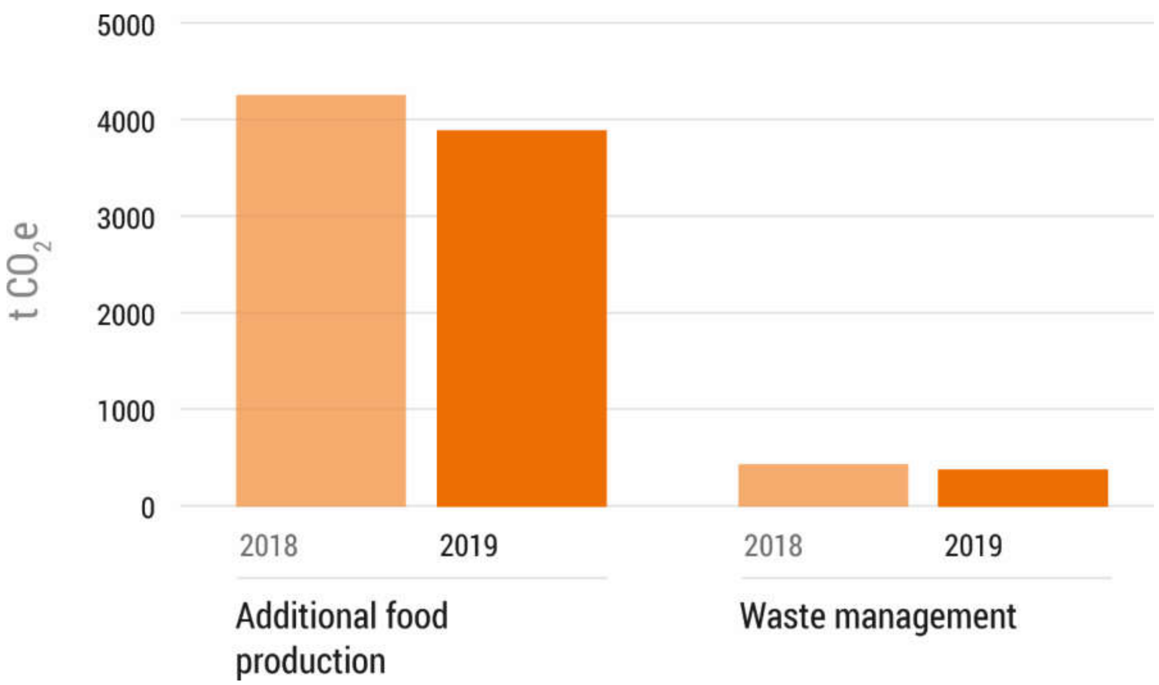

Figure S2: Comparison of greenhouse gas emissions by category in a scenario "without the action of the Food Bank of Navarra" between 2018 and 2019 (tonnes of CO_2e).

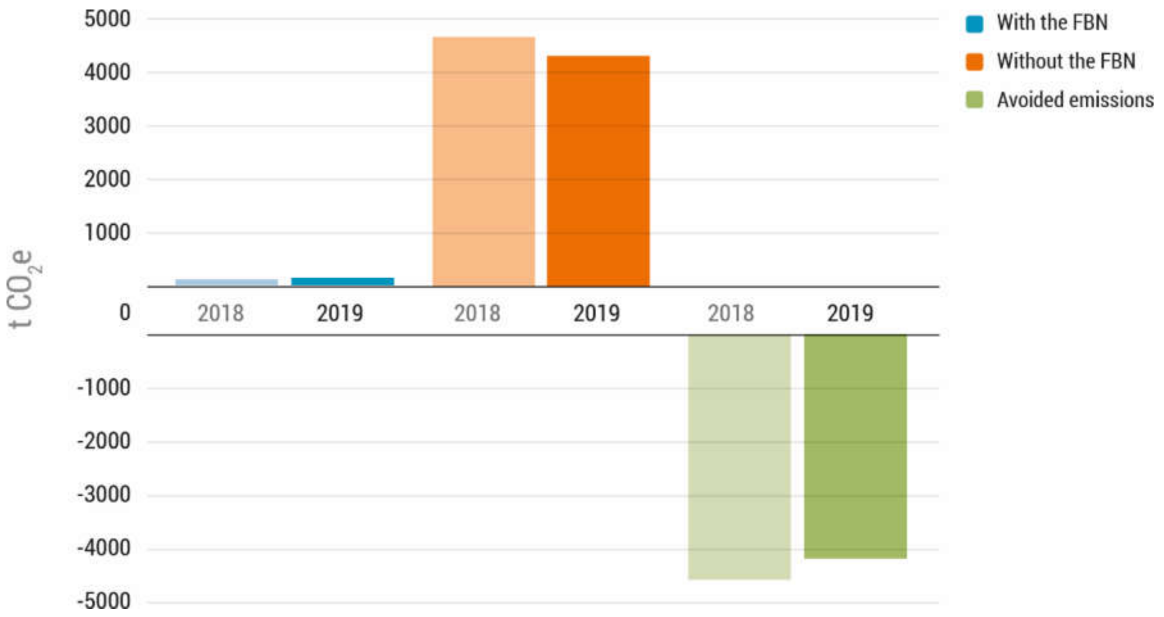

Figure S3: Comparison of total greenhouse gas emissions in the scenarios "with" and "without" the action of the Food Bank of Navarra, and emissions avoided by the Food Bank of Navarra between 2018 and 2019 (tonnes of CO_2e).
